# A Pilot Study Evaluating the Feasibility of Testing for an Acute Impact of Human Exposure to a Power‐line Frequency Magnetic Field on Blood Cortisol and Thyroid‐Stimulating Hormone

**DOI:** 10.1002/bem.22426

**Published:** 2022-11-20

**Authors:** Alexandre Legros, Michael Corbacio, Sébastien Villard, Martine Souques, Jacques Lambrozo

**Affiliations:** ^1^ Bioelectromagnetics and Human Threshold Research Group, Imaging Department Lawson Health Research Institute Ontario London Canada; ^2^ Departments of Medical Biophysics, Medical Imaging and School of Kinesiology Western University Ontario London Canada; ^3^ EuroMov Digital Health in Motion, Université de Montpellier IMT Mines Ales Montpellier France; ^4^ Division Neuromodulation R&D EuroStim Montpellier France; ^5^ Service des Études Médicales EDF Levallois‐Perret France

**Keywords:** magnetic field, extremely low frequency, cortisol, thyroid‐stimulating hormone, pilot feasibility study, human study

## Abstract

Numerous studies have been carried out on the potential effects of an extremely low frequency (ELF—0–300 Hz) magnetic field (MF) on human health. However, there is limited data on the effect of a high exposure level to ELF MFs for a prolonged period. Therefore, the objective of this pilot work was to demonstrate the feasibility of a study evaluating the stress hormone concentrations resulting from a 10‐min exposure to a 60 Hz MF of several tens of thousands of µT. In this pilot study, human volunteers were thus exposed for the first time to a 60 Hz, 50 mT MF for a duration of 10 min. Stress hormone levels were measured before (once), during (twice) and after (once) this 10‐min exposure period. The small sample size (*n* = 5) did not allow to conduct standard inferential statistical tests and no conclusion regarding the exposure effects can be drawn. However, this study demonstrates the feasibility of using a simple blood testing material in a protocol testing for the effect of a 10‐min exposure to a high MF level in healthy human volunteers. © 2022 Bioelectromagnetics Society.

## INTRODUCTION

Visual flickering perceptions resulting from the exposure to an intense extremely low frequency (ELF—0–300 Hz) magnetic field (MF) have been described since the end of the 19th century by D'Arsonval [1896]. These have been called magnetophosphenes. They have since been studied in seminal publications [Lövsund et al., 1979; Lövsund et al., 1980], from which they have been selected as the scientific basis for international recommendations to ELF MF exposures [Saunders and Jefferys, 2007; ICNIRP, 2010]. However, uncertainties related to electrophosphene (i.e. resulting from electric stimulations) and magnetophosphene perception thresholds have been highlighted [Kavet et al., 2008]. Over the last few years, our research group at the “Lawson Health Research Institute” has focused on more precisely establishing the magnetophosphene perception threshold through experimental studies, carried out in healthy human volunteers exposed to up to 50 mT at 50 and 60 Hz [Legros, 2015]. While this type of study is essential to establish neuro‐physiological response thresholds, it does not allow the identification of potentially lasting biological effects persisting a few min after the exposure has ended. This is the main motivation behind this pilot study: showing the feasibility of a study identifying whether prolonged exposure (in the order of minute) to a high level of ELF‐MF can induce post‐exposure biological changes in selected blood parameters.

The fact that cortisol (compound F) is a sensitive blood marker resulting from acute or chronic stress motivated its use in the present pilot work. Is it possible for an ELF‐MF to induce acute stress response as measured through plasma cortisol concentration? Cortisol secretion peaks between 6:00 am and 8:00 am, then decreases to almost zero in the middle of the night, before increasing again to reach a new peak the next morning [Selmaoui and Touitou, 2003]. For this reason, plasma cortisol needs to be measured in the morning, around 8:00 am.

Thyroid‐stimulating hormone (TSH) secretion is another blood stress marker we selected for this pilot work. TSH does not follow the same dynamics as cortisol. Like cortisol, it is subject to a circadian rhythm, but it is pulsatile, with around 12 pulses of 0.5 mIU/L in 24 h. Above all, TSH is not influenced by external factors that can generate acute stress, such as physical exercise, outside temperature or posture. Finally, unlike cortisol secretion, TSH is controlled by negative feedback from thyroid hormones, dopamine and glucocorticoids, and stimulated by noradrenaline [Herbomez, 2014]. All these physiological factors allow TSH to be reported as a hormone *a priori* independent to possible acute stress in comparison to cortisol.

Previous work on blood parameters in humans were mainly carried out on electricity utility company workers at levels not exceeding 1 mT and they showed no difference between exposed and control [World Health Organization, 1984, 1987; Gamberale et al., 1989]. Here we are exploring a flux density 50 times higher in the same frequency range.

## METHODS

The fingertip blood sampling method developed by HemaSpot (http://www.spotonsciences.com/hemaspot-hf/) enabled this feasibility study on five adult volunteers (two women and three men, 38 ± 16 years old) testing for cortisol and TSH concentrations (other stress hormones were not available at the time of the study).

**Fig. 1:**
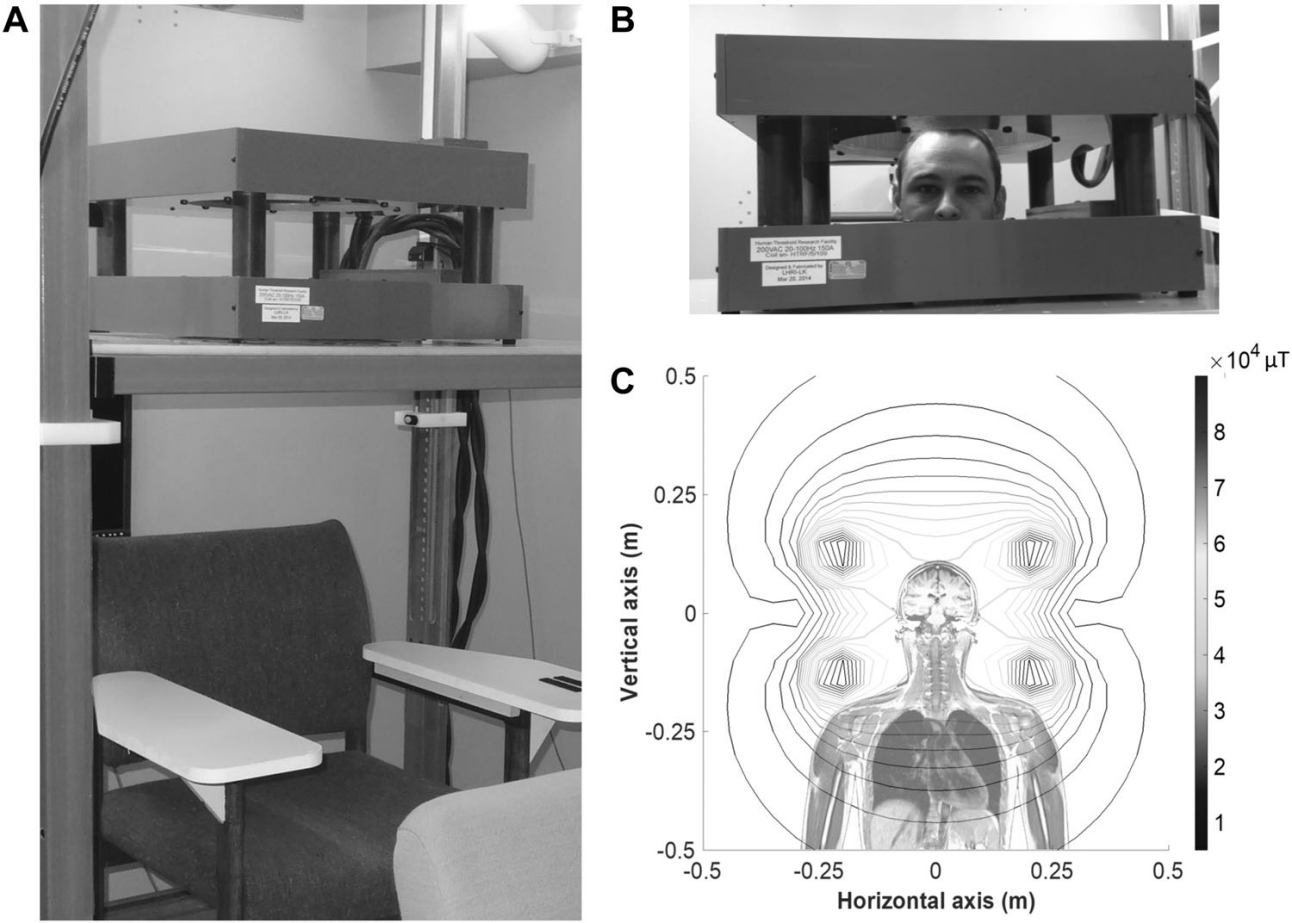
**A**: The coil system mounted on the vertically adjustable elevator platform. **B**: Participant with their head in the coils, positioned within the 50 mT homogeneity region. **C**: Theoretical magnetic field distribution generated by the coils, with 5 mT contour lines (the color scale on the right is expressed in 10^4^ μT). The thicker inner contour line represents the 50 ± 5 mT homogeneity region.

The exposure system consisted in a Helmholtz‐like assembly of two circular coils of 99 turns of hollow copper wire each (allowing for water cooling circulation—35.6 cm inner diameter and 50.1 cm outer diameter). The wire of these coils is glued with very resistant epoxy as one solid bloc, which avoids the production of vibration sounds. The two coils are mounted vertically (separated by 20.6 cm) as shown in Fig. 1**A**, allowing the volunteer to put his head in the center of the coils. This exposure system, powered with MTS MRI gradient amplifiers (MTS Automation Model No. 0105870, Horsham, PA, USA) and driven by a LabView program (National Instruments, Austin, TX, USA) is described in previous work [Bouisset et al., 2020]. A more detailed dosimetry has been conducted and will be published in an upcoming article on magnetophosphenes thresholds in humans, which shows low estimated induced E‐field levels in the center of the brain (in the order of 0 to 0.2 V/m) and maximum values on the cortical layers (maximum values in the order of 1.3 V/m at 60 Hz). Note that the amplifiers cabinet is placed in a separate soundproof room, which prevents any sound to get to the experimental room. The hollow copper wire allows for the circulation of cooled water, hence preventing the coils from overheating during the 10‐min exposure to a vertical 60 Hz MF at 50 mT. In order to confirm that heating at the level of the volunteer's head would not be a confounding factor, we measured the heat at the surface of a phantom head (a watermelon) placed in the center of the coils for a period of 20 min. The results show that despite a limited heating at the contact of the coils, no temperature increase was measured at the head level.

Each volunteer participated in two experimental sessions during which their entire head was placed inside the MF exposure system (Fig. 1**B**). One of the experimental sessions was a control session (no active exposure) and the other was an active exposure session with a 10‐min exposure to a 50 mT MF at 60 Hz. Despite their silent design, the coils made a slight buzzing sound at this field level, which was canceled by volunteers wearing earplugs. In order to respect the cortisol cycle and to have reliable results between sessions, the two sessions were carried out at the same time of the day (8:00 am), on 2 different days. This pilot work was conducted as part of the HSREB 1886 ethics protocol approved by Western University.

## RESULTS

**Fig. 2:**
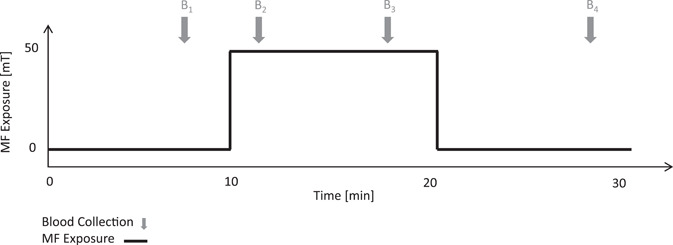
Experimental procedure timeline. An experimental session lasted 30 min total, the MF exposure started at minute 10 and ended at minute 20. The MF is only present during the real condition and not in the control condition. The gray arrows mark the start of blood collection: at the 7th minute of the session (i.e. 3 min prior to the onset of the MF), at the 12th minute of the session (i.e. 1 min after the start of the MF), at the 17th minute of the session (i.e. 7 min after the start of the MF) and at the 27th minute of the session (i.e. 7 min after the end of the MF). The black line indicates the MF flux density (0 or 50 mT). MF, magnetic field.

Blood samples were taken using HemaSpot (CoreMedica Laboratories, Lee's Summit, MO) before the exposure onset (3 min before the MF starts), during the exposure (2 and 7 min after the exposure has started) and after the end of the exposure (7 min after the exposure ends, see Fig. 2 for a timeline). The blood test analyses were sent and conducted in the CoreMedica certified laboratory (blinded), the results were then sent back to us.

**Fig. 3:**
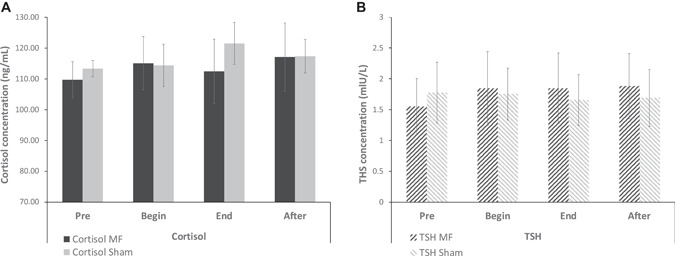
**A**: Mean cortisol levels of the five healthy participants in each exposure conditions. The error bars represent the Standard Error of the Mean. **B**: Mean TSH levels of the five healthy participants in each condition. The error bars represent the Standard Error of the Mean. Pre: before the exposure, at 7 min (after the beginning of the experimental session); Begin: during the exposure, at 12 min; End: during the exposure, at 17 min; After: after the exposure, at 27 min). TSH, Thyroid‐stimulating hormone.

The cortisol and TSH descriptive results Fig. 3 indicate that the participants had blood concentration values within normal limits for both morning pre‐exposure measurements. Indeed, at 8:00 am normal cortisol levels range between 50 and 230 ng/ml, which is consistent with our measurements. Regarding normal TSH values, they are reported to be between 0.4 and 4 mIU/L, which is again consistent with our measurements. Although no statistical tests have been conducted due to the very small sample size (it was not the intent of this pilot feasibility protocol to allow for inferential statistics to be conducted), no difference between the control and the exposure sessions is suggested by the descriptive statistics presented in Fig. 3. We need to mention that the assumption of measurement stability in subjects with cortisol or TSH levels reflecting a pathology cannot be ruled out.

## DISCUSSION

Although this protocol was not designed to study magnetophosphenes perception, all the volunteers reported seeing them within the first few min of exposures. This thus made possible for the volunteers to distinguish between the control and the exposure sessions. It is important to note that it is impossible to prevent the appearance of these visual phenomena at this level of ELF‐MF flux density at 60 Hz. Although the volunteers were familiar with magnetophosphenes perception (they had already participated in a research protocol testing for magnetophosphenes thresholds) and that they were likely not influenced by magnetophosphenes perception, it cannot be excluded that this might be a confounding experimental factor. However, blood samples analyses were carried out without knowledge of the presence or absence of exposure by the laboratory technician, which blinds the analysis. Interestingly, magnetophosphenes were not visible throughout the entire exposure period, suggesting a possible habituation of the visual system to the exposure even at this high level. This adaptation phenomenon had already been observed by Lövsund [Lövsund et al., 1980] but requires further investigations since it is poorly documented.

## CONCLUSION

Note again that the objective of this work is not to report statistical effects of the 10‐min MF exposure but to demonstrate the feasibility of this procedure on a larger group of volunteers in the future. This pilot study actually shows the feasibility of fingertip blood samples analyses. It is important also to report that, even though this is the longest human continuous exposure period to an ELF, 50 mT MF, no participants reported any adverse effects. In conclusion, this work used the highest MF exposure level at power‐frequency and it demonstrated the protocol feasibility to study the potential MF effects on human blood biochemistry.

## References

[bem22426-bib-0001] Bouisset N , Villard S , Legros A . 2020. Human postural control under high levels of extremely low frequency magnetic fields. IEEE Access 8:101377–101385.

[bem22426-bib-0002] D'Arsonval' . 1896. Dossier d'Arsonval (Je sais tout + CR de l'académie des sciences).

[bem22426-bib-0003] Gamberale F , Olson BA , Eneroth P , Lindh T , Wennberg A . 1989. Acute effects of ELF electromagnetic fields: a field study of linesmen working with 400 kV power lines. Occup Environ Med 46:729–737. 10.1136/oem.46.10.729 PMC1009855 2818960

[bem22426-bib-0004] Herbomez M . 2014. Hormone thyréostimulante (TSH) [90‐10‐0555‐A]. Biologie Médicale. Paris: Elsevier.

[bem22426-bib-0005] ICNIRP . 2010. ICNIRP statement on the “Guidelines for limiting exposure to time‐varying electric, magnetic, and electromagnetic fields (up to 300 GHz)”. Health Phys 97:257–258. 10.1097/HP.0b013e3181aff9db 19667809

[bem22426-bib-0006] Kavet R , Bailey WH , Bracken TD , Patterson RM . 2008. Recent advances in research relevant to electric and magnetic field exposure guidelines. Bioelectromagnetics 29:499–526. 18618584 10.1002/bem.20423

[bem22426-bib-0007] Legros A . 2015. Magnetophosphenes percepion threshold and EEG response in humans exposed to 20, 50, 60 and 100 Hz MF up to 50,000 μT. In: Annual meeting of the Bioelectromagnetics Society and the European Bioelectromagnetics Association, BioEM, Asilomar. California, USA.

[bem22426-bib-0008] Lövsund P , Oberg PA , Nilsson SE . 1979. Influence on vision of extremely low frequence electromagnetic fields. Industrial measurements, magnetophosphene studies volunteers and intraretinal studies in animals. Acta Ophthalmol 57:812–821. 525304

[bem22426-bib-0009] Lövsund P , Öberg PÅ , Nilsson SEG , Reuter T . 1980. Magnetophosphenes: a quantitative analysis of threshold. Med Biol Eng Comput 18:326–334. 6968384 10.1007/BF02443387

[bem22426-bib-0010] Saunders RD , Jefferys JGR . 2007. A neurobiological basis for ELF guidelines. Health Phys 92:596–603. 17495661 10.1097/01.HP.0000257856.83294.3e

[bem22426-bib-0011] Selmaoui B , Touitou Y . 2003. Reproducibility of the circadian rhythms of serum cortisol and melatonin in healthy subjects: a study of three different 24‐h cycles over six weeks. Life Sci 73:3339–3349. 14572876 10.1016/j.lfs.2003.05.007

[bem22426-bib-0012] World Health Organization . 1984. Extremely Low Frequency (ELF) Fields. Geneva: World health organization.

[bem22426-bib-0013] World Health Organization . 1987. Magnetic Fields. Geneva: World health organization.

